# Tailored Drug Release with Improved Solubility via Spray Drying and Functional Ready-to-Fill Capsules

**DOI:** 10.3390/pharmaceutics18070838

**Published:** 2026-07-09

**Authors:** Kristina Vlahovic, Lilla Dorottya Kóró, Bence Dávid Tóth, Dávid Hamar, Nikolett Kállai-Szabó, András József Laki, István Antal, Miléna Lengyel

**Affiliations:** 1 Department of Pharmaceutics, Semmelweis University, Hőgyes Endre Street 7, 1092 Budapest, Hungary; vlahovic.kristina@phd.semmelweis.hu (K.V.); koro.lilla.dorottya@semmelweis.hu (L.D.K.); toth.bence@semmelweis.hu (B.D.T.); hamar.david@semmelweis.hu (D.H.); kallai.nikolett@semmelweis.hu (N.K.-S.); 2 Center for Pharmacology and Drug Research & Development, Semmelweis University, Üllői Street. 26, 1085 Budapest, Hungary; 3 Faculty of Information Technology and Bionics, Pázmány Péter Catholic University, Práter Street 50/A, 1083 Budapest, Hungary; laki.andras1@semmelweis.hu; 4 Department of Biophysics and Radiation Biology, Semmelweis University, Tűzoltó Street 37-47, 1094 Budapest, Hungary

**Keywords:** spray-drying, tailored drug release, modified-release, solubility enhancement, albendazole, tartaric acid, functional capsules, microenvironmental pH, experimental design, solid dispersions

## Abstract

**Background/Objectives:** To improve the potential therapeutic efficacy of albendazole (ABZ), its low solubility in alkaline environments should be addressed. Spray drying has been widely applied to increase the solubility of poorly soluble drugs. Eudragit^®^ E PO (EudE) has previously been used to increase the solubility of weakly basic drugs, alone and in combination with organic acids. In the present work, we aimed to demonstrate that EudE and tartaric acid (TA) significantly increase the solubility of ABZ via spray drying. Furthermore, a colon-delivery formulation was applied to deliver ABZ at pH 7.2. **Methods:** This study developed spray-dried formulations of ABZ-TA-EudE. The formulation with the best solubility profile was selected and loaded into the EUDRACAP^®^ colon to achieve colon-delivery of the ABZ formulation with enhanced solubility at basic pH. The physicochemical characteristics and in vitro drug release were evaluated and compared with those of the respective physical mixtures. **Results:** The addition of TA further enhanced the solubility of ABZ-EudE formulations. Both EudE and TA significantly increase ABZ solubility. The ternary ABZ-TA-EudE formulation with the best solubility, when delivered into the EUDRACAP^®^ colon, showed enhanced ABZ release at pH 7.2. Enhanced in vitro drug release at basic pH and physicochemical characteristics were observed for the spray-dried formulation of ABZ-TA-EudE compared with the corresponding physical mixture and the ABZ powder. **Conclusions:** TA and EudE significantly enhance the solubility of the weakly basic drug, ABZ, in a spray-dried formulation. Furthermore, the application of EUDRACAP^®^ colon is effective for colon-delivery of ABZ, as it ensures ABZ release at pH 7.2, thereby providing enhanced formulation’s solubility at its site of action.

## 1. Introduction

Albendazole (ABZ) is an antihelmintic drug [1] that has also been investigated as an antitumor agent in colon cancer [2]. ABZ has been classified as BCS II/IV (BCS-Biopharmaceutical Classification System), indicating low solubility and high/low permeability [3]. The poor aqueous solubility limits its dissolution in biological fluids, leading to low absorption and, consequently, low bioavailability. Furthermore, ABZ solubility is pH-dependent, with higher solubility at lower pH [4]. Thus, local delivery of ABZ to the lower gastrointestinal tract, with increased solubility, would optimize its bioavailability and consequently the therapeutic effect of ABZ for colon diseases [5].

Previous studies have investigated various formulations to improve the solubility of weak bases, such as ABZ. Fülöp et al. formulated nanosuspensions containing micronized ABZ with an enhanced dissolution rate compared to those containing unmilled ABZ and pure powder. The improved dissolution performance was due to milling ABZ and the presence of the surfactant Polysorbate 80, which is necessary for stabilizing the nanosuspension [6]. Guo et al. aimed to develop nanosuspensions to improve the solubility of ABZ, using EUDRACAP^®^ capsules for colon delivery to maximize release at the site of action. An increase in solubility at the targeted site of action can improve bioavailability and, therefore, reduce the dose required for therapeutic efficacy [5].

Another effective method for enhancing ABZ solubility is the formulation of solid dispersions [7,8]. However, to achieve a greater increase in dissolution rate, stronger molecular-level mixing with the polymer in the solid dispersions is required [7]. Almotairy et al. showed that incorporating pH-modifiers into telmisartan-polymer solid dispersions improved the release of telmisartan by creating a suitable microenvironmental pH [9]. Previous studies have suggested the use of weak organic acids in solid dispersions, where ionic interactions between the acid and the API led to a solubility increase [10].

In our previous work, TA, as a pH modifier, was used in a controlled-release dosage form to enhance the dissolution rate of ABZ by creating an acidic microenvironmental pH. A study by Fung et al. applied organic acids to co-amorphous systems with a basic drug, showing that these systems exhibited a higher dissolution rate than the drug alone due to the acidic microenvironmental pH created by the organic acids and due to the size and morphology of the spray-dried particles [11]. Furthermore, Mild et al. demonstrated that incorporating tartaric acid into the ternary organic acid-drug-polymer system led to the formation of an ionically crosslinked polymer-acid network that controls dissolution performance [12].

Solid dispersions of a poorly water-soluble active pharmaceutical ingredient (API), of an amorphous or crystalline morphology, can be produced from a carrier, either amorphous or crystalline [13,14]. The selection of the excipient can significantly influence the drug-release pathway from a solid dispersion. Dimethylaminoethyl methacrylate-copolymer Eudragit^®^ E PO (EudE) is a cationic copolymer soluble in an acidic gastric pH, because of the protonation of dimethylamino groups [15,16]. Several studies have shown that the EudE polymer has been shown to increase the solubility of poorly water-soluble drugs in solid dispersions, such as rosuvastatin [13]. Maghsoodi et al. showed that EudE forms specific interactions (hydrogen bonds) with dipyridamole and that the presence of EudE in the solution further increased the released drug concentration from the tartaric acid-based formulation, by inhibiting precipitation [17]. Another study by Maghsoodi et al. suggested that adding tartaric acid (TA) to EudE-dipyridamole systems increased the specific drug-polymer interactions and drug solubility [18].

Targeted colon delivery of ABZ has shown advantages, including higher solubility and, consequently, greater bioavailability [5]. Our previous study used a specific combination of Eudragit^®^ polymers to provide site-specific delivery of ABZ to higher pH levels in a modified-release multiparticulate dosage form with functional tartaric acid pellet cores. Eudragit^®^ FS effectively prevented ABZ release at gastric pH, thereby maximizing drug release in a neutral to slightly alkaline environment [19].

The present study aimed to improve the solubility of ABZ by formulating a solid dispersion via spray drying, a common method for producing spherical, smooth particles with increased dissolution rate [20]. EudE and TA have been used to improve the solubility of ABZ. EudE has previously been described as a component that can improve the solubility of both acidic and basic drugs via nonspecific hydrophobic interactions [21].

We formulated solid dispersions of ABZ-EudE only, and ABZ-EudE-TA of different ratios to investigate thermodynamic solubility. Our hypothesis was that, despite the absence of specific drug-polymer interactions, EudE increases ABZ solubility, and that this solubility reaches a plateau at a specific ratio. Furthermore, the aim was to incorporate TA, which was expected to further enhance the solubility of ABZ-EudE formulations by increasing specific interactions and promoting the amorphization of the active ingredient during spray drying. To support this hypothesis, the interactions between the drug and the polymer, and between the drug, polymer, and the organic acid, were investigated. These investigations aimed to show that, in addition to hydrophobic interactions, specific interactions occur when TA is present.

Our further hypothesis was that ABZ could reach a relatively high concentration at pH 7.2. To reach this goal, the solid dispersion ABZ-TA-EuE with the highest solubility was loaded into EUDRACAP^®^ colon-targeted, ready-to-fill capsules to achieve a site-specific delivery of ABZ at pH 7.2.

EUDRACAP^®^ colon capsules are precoated HPMC capsules that are combined with a pH-dependent polymer, which remarkably simplifies the production. To the best of our knowledge, EUDRACAP^®^ colon capsules have not been used previously for colon delivery of EudE-TA-based spray-dried powders with improved solubility. The novelty of the formulation, furthermore, lies in the tailored release of the ABZ with a preparation produced from an ionically crosslinked drug-polymer-organic acid system. This system is characterized by specific and non-specific interactions and a suitable microenvironmental pH, which influence the amorphousness of the sample and drug release in combination with an easy-to-fill modified-release capsule shell. To evaluate the performance of the capsule shells, the in vitro drug release from EUDRACAP^®^ colon capsules was compared with that from hydroxypropyl methylcellulose (HPMC) capsules. Furthermore, to demonstrate the advantage of spray drying in improving drug solubility, the physicochemical properties and in vitro drug release of the spray-dried formulations were compared with those of the respective physical mixtures (PMs).

## 2. Materials and Methods

### 2.1. Materials

**Figure 1:**
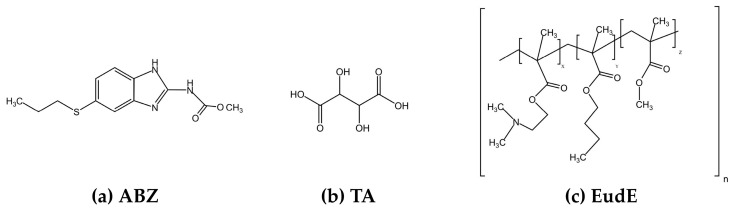
Chemical structures of (**a**) albendazole (ABZ); (**b**) tartaric acid (TA), and (**c**) Eudragit^®^ E PO (EudE).

Albendazole EP (ABZ) (micronized) (SeQuent Scientific Ltd., Bilekahalli, India) was used as a model drug. Dimethylaminoethyl methacrylate-copolymer (Eudragit^®^ E PO, Evonik, Essen, Germany)—abbreviated as EudE—was used as a carrier in the solid dispersion. Tartaric acid (Molar Chemicals Ltd., Halásztelek, Hungary)—abbreviated as TA—was used further to improve the solubility of the EudE-based solid dispersion. The ready-to-fill EUDRACAP^®^ colon capsules were kindly provided by Evonik Operations GmbH (Essen, Germany). Hydroxypropyl methylcellulose (HPMC) capsules (Capsugel^®^ Vcaps^®^ Plus), used for comparison with EUDRACAP^®^ colon, were obtained from Lonza (Basel, Switzerland). The chemical structures of ABZ, TA and EudE are given in Figure 1. Sodium chloride, hydrochloric acid 37% (*w*/*w*), potassium dihydrogen phosphate and sodium hydroxide (Molar Chemicals Ltd., Halásztelek, Hungary) were used to prepare the pharmacopoeial buffers for further studies.

### 2.2. Preparation of ABZ-Loaded Solid Dispersions by Spray Drying

ABZ-loaded solid dispersions (SDs) were prepared by dispersing 2 g of ABZ in the previously prepared solution of TA and EudE in water, under magnetic stirring using a heatable magnetic stirrer MS-H-S10 (DLAB Instruments Ltd., Beijing, China) and magnetic stirrer bars at 25 ± 0.5 °C for 24 h, to achieve the following ABZ:TA:EudE ratios (1:0:0; 1:0:1; 1:0:2; 1:0:2.5; 1:0:4.5; 1:3:0; 1:6:0; 1:3:1; 1:6:1; 1:3:2 and 1:6:2). The detailed formulations are summarized in Table 1. The volume of water used for all the dispersions was the amount required to achieve a dispersion concentration of 18% (*w*/*v*). The compositions containing only EudE (1:0:0, 1:0:1, 1:0:2, 1:0:2.5, and 1:0:4.5) were used to determine the ABZ:EudE ratio at which the solubility increase due to the polymer reached a plateau. All of the prepared dispersions were spray-dried after 24 h of mixing using the ProCepT Trix 4M8 Spray dryer (ProCepT, Zelzate, Belgium). The batch size was formulation-dependent, ranging from 2 g to 18 g of theoretical dry solids, assuming 2 g of ABZ and 18% (*w*/*v*) were held constant. A 0.8 mm nozzle with a nozzle air pressure of 1.8 ± 0.2 bars was used. The feed rate was 1.5 g/min. The air speed was 0.3 m^3^/min, while the inlet temperature was adjusted to maintain an outlet air temperature of 30 ± 10 °C. The homogeneity of the solid dispersions was assessed by determining the ABZ content in 6 parallel samples. The standard deviation was less than ±5%. The average water content for 1:6:2 spray-dried solid dispersion was 0.845 ± 0.011, determined by Karl-Fisher 787 KF Titrino (Metrohm AG, Herisau, Switzerland).

### 2.3. Preparation of Physical Mixtures

Physical mixtures of ABZ, TA, and EudE were prepared by geometric mixing with a mortar and pestle (5 min), after having passed the components through a stainless steel sieve (d = 0.18 mm) (Retsch GmbH, Retsch-Verder Company, Vleuten, The Netherlands), triturating to achieve the same ratios as those given for the spray-dried formulations in Table 1. All blends were prepared with a batch size of 20 g. The homogeneity of the physical mixtures was controlled by determining ABZ content in 6 parallel samples. The standard deviation was less than ±5%. The average water content of the 1:6:2 physical mixture was 0.164 ± 0.025, determined using a Karl-Fisher 787 KF Titrino (Metrohm AG, Herisau, Switzerland).

### 2.4. Scanning Electron Microscope (SEM) Imaging

The SEM images were processed using a Hitachi TM4000 Plus II Tabletop Scanning Electron Microscope (Hitachi, Tokyo, Japan). The samples were prepared and fixed to double-sided conductive tape. The images were acquired in standard vacuum mode at an accelerating voltage of 5 kV, lens mode 3, and a mixed detector. The SEM image of the spray-dried sample was compared to the respective physical mixture.

### 2.5. Thermodynamic Solubility Studies

To investigate the increase in solubility in phosphate buffer pH 6.8 (Ph. Eur. 11), with the ionic strength (I = 0.10 M) in the presence of TA and EudE, the ratio in the ABZ-TA-EudE ternary formulations varied: 1:0:0; 1:0:1; 1:0:2; 1:3:0; 1:6:0; 1:3:1; 1:6:1; 1:3:2 and 1:6:2. The spray-dried solid dispersions and the corresponding physical mixtures containing an excess amount of ABZ powder (10 mg) were transferred to 10 mL volumetric flasks, filled with phosphate buffer (pH 6.8, Ph. Eur. 11). The volumetric flasks were mixed using a heatable magnetic stirrer MS-H-S10 (DLAB Instruments Ltd., Beijing, China) and magnetic stirrer bars at 25 ± 0.5 °C for 24 h. The samples containing ABZ suspensions were filtered with a 0.22 µm pore-sized hydrophilic PTFE syringe membrane filter (Labex FilterBio Membrane Co., Ltd., Nantong, China). The amount of ABZ was calculated from linear calibration in the dissolution medium, based on three parallel measurements, and determined by spectrophotometry at the absorption maximum of albendazole (λ_max_ = 295 nm) using an Agilent 8453 spectrophotometer (Agilent Technologies Inc., Santa Monica, CA, USA). The pharmacopoeial buffer had a nominal pH of ±0.05.

### 2.6. Statistical Evaluation of Thermodynamic Solubility

The effect of TA and EudE on solubility increase was evaluated using a two-factor, three-level experimental design based on thermodynamic solubility study measurements (solubility in µg/mL). The two variables and their coded values for the experimental setup are summarized in Table 2. The values of the reported ratios of TA and EudE indicated in Table 2 are the ratios compared to 1 part of ABZ in all the compositions. The data were evaluated and graphically represented using TableCurve^®^ 3D v4.0 Software (Systat Software Inc., London, UK).

A second-order polynomial model was used to determine the quantitative influence of the independent variables on the response variable y (solubility).

(1) $$y=b_{0}+b_{1}x_{1}+b_{2}x_{2}+b_{11}x_{1}^{2}+b_{22}x_{2}^{2}+b_{12}x_{1}x_{2}$$

Here, x_1_ and x_2_ represent the factors under investigation, while the parameters b_0_, b_1_, b_2_, b_11_, b_22_ and b_12_ represent the linear (b_1_, b_2_), nonlinear (b_11_, b_22_) and interaction (b_12_) effect coefficients. This model enabled the systematic investigation of the relationships between the number of excipients used in the formulation and ABZ solubility.

### 2.7. Fourier Transform Infrared (FT-IR) Spectroscopy

Fourier transform infrared (FT-IR) spectroscopy was performed using a FT-IR spectrophotometer equipped with a single reflection accessory (FT/IR-4200 with ATR PRO470-Hin, Jasco Products Company, Oklahoma City, OK, USA) in the spectral range of 400 to 4000 cm^−1^. After performing 100 scans, the measurements were evaluated using the OriginPro^®^ 2026 trial version, which enabled precise peak identification.

### 2.8. Differential Scanning Calorimetry (DSC)

DSC measurements were performed on a Discovery DSC 2500 (TA Instruments, New Castle, DE, USA) under flowing nitrogen (50 mL/min cell purge and 300 mL/min base purge) with a heating rate of 5–10 °C/min between 40 °C and 250 °C. Solid samples in the 3.0–8.0 mg range were accurately weighed into Tzero hermetic pans. Before the measurements, the melting points of the samples were determined using a BÜCHI Melting Point B-450 apparatus (Büchi Labortechnik, Flawil, Switzerland). The accurate weighing normalized the results.

### 2.9. Powder X-Ray Diffractometry (P-XRD)

The measurements aimed to determine the extent of amorphization of ABZ in binary (ABZ-EudE) and ternary (ABZ-TA-EudE) spray-dried solid dispersions.

P-XRD diffractograms of pure drug, API, EudE, TA, and their solid dispersions were obtained by XPRD (X’pert Pro MPD X-ray diffractometer, Malvern PANalytical, Malvern, UK), using a 2θ range of 2.00–39.99 for the characterization of the powders. The X-ray power was set to 45 kV and 40 mA. Samples were prepared following a standard procedure. The powder samples were carefully loaded into the sample holder without prior sieving or grinding. Excess material was then gently removed using a paper card, leaving a relatively smooth sample surface. The peaks were measured over a 2θ range of 2.00–39.99 for powder characterization and evaluated using the OriginPro^®^ 2026 trial version.

The crystallinity index (CI) was calculated according to the following formula (Equation (2)) [10]:

(2) $$\mathrm{Crystallinity}\;\mathrm{index}\;(\%)\;=\frac{\mathrm{area}\;\mathrm{of}\;\mathrm{selected}\;\mathrm{crystalline}\;\mathrm{peaks}}{\mathrm{total}\;\mathrm{area}\;\mathrm{of}\;\mathrm{diffraction}}\cdot 100$$

The peaks were measured and evaluated with OriginPro^®^ 2026 trial version. The method aimed to determine the extent of amorphization of ABZ in spray-dried solid dispersions and in the respective binary EudE-solid dispersions.

### 2.10. In Vitro Drug Release Tests and Release Kinetics by Weibull Model Fitting

A sample of the spray-dried composition with the greatest increase in solubility (1:6:2 SD) was loaded into EUDRACAP^®^ colon and HPMC capsules. Furthermore, the respective physical mixture (1:6:2 PM) and ABZ powder were also loaded into the EUDRACAP^®^ colon to compare them with the spray-dried sample. The dissolution test for each type of capsule was carried out in a 500 mL dissolution medium of pharmacopoeial compositions (Ph. Eur. 11): pH 1.5 hydrochloric acid (I = 0.10 M) for 1 h, in phosphate-buffered solution pH 4.5 (I = 0.10 M) for the next 3 h, and finally in phosphate-buffered solution pH 7.2 (I = 0.12 M) for the next 4 h, in accordance with the recommendations of Pharmacopeia [22]. The amount of powder used for dissolution contained 10 mg of ABZ, as reported by Guo et al. [5]. The USP paddle method with 100 rpm at 37 ± 0.5 °C was used (Hanson SR8-Plus™ Dissolution Test Station, Teledyne Hanson Research, Chatsworth, CA, USA). At predetermined time points, 5 mL samples were withdrawn. The concentration of released ABZ was measured spectrophotometrically at λ_max_ = 295 nm (Agilent 8453 spectrophotometer; Agilent Technologies Inc., Santa Monica, CA, USA). The in vitro drug release of ABZ solid dispersion (1:6:2 SD), ABZ powder and the respective physical mixture (1:6:2 PM) was performed in the following set of conditions: pH 1.5, 1 h, pH 4.5, 1–4 h and pH 7.2 up to 8 h, while the in vitro drug release from HPMC capsules reached 100% already at pH 1.5. The volume of the withdrawn samples was replaced with an equal amount of pre-warmed dissolution medium. The dissolution test was performed in triplicate.

The ionic strength of the respective buffers was calculated according to the following equation (Equation (3)) (I is the ionic strength, j is the number of different ions in the solution, Z_i_ is the charge of ion i, C_i_ is the molar concentration of ion i to j [23].

(3) $$I=\frac{1}{2}\sum_{i}^{j}\left(c_{i}\cdot z_{i}^{2}\right)$$

The release mechanism kinetics were successfully described using cumulative dissolution profiles fitted by the Weibull model by many authors [24,25,26].

This function is particularly well-suited to characterizing drug release processes where there is no uniform curve shape. The Weibull equation is used to fit the release data based on the following factors (Equation (4)):

(4) $$M_{t}\;={\;M}_{\infty \;}\left[1-\exp{\left(-\frac{t\;-t_{0}}{\tau_{d}}\right)}^{\beta }\right]$$

*M_t_* represents the proportion of the active substance released up to time *t*, starting at *t*_0_, the initial time. $M_{\infty \;}$ represents the maximum release amount, β is the shape factor of the function, and *t*_0_ represents the lag time. τ_D_ stands for the characteristic time parameter (the time at which 63.2% of the active substance is released).

The fitting of the distribution function was performed using the Analytic Solver function of Microsoft Excel (Microsoft Office 365), GRG Nonlinear Engine based on the sum square minimization with the following constraints: all factors are assumed nonnegative, t_0_ is greater than the release value of the first timing point. All percentage release values below 5% were considered as 0 for the evaluation of the lag-time.

### 2.11. Microenvironmental pH Studies (Micro pH)—By Slurry Method

Microenvironmental pH (Micro pH) was studied using the slurry method described by Fung et al. [11]. The slurry pH is intended to represent the microenvironmental pH [11,27,28]. Solid dispersions containing 5 mg of ABZ were added to High Performance Liquid Chromatography (HPLC) vials, and 1 mL of distilled water was added. The slurries were then allowed to settle at 25 ± 0.5 °C for 24 h, after which the pH was measured using a Mettler-Toledo Seven Compact micro pH/Ion metre (Mettler-Toledo International Inc., Columbus, OH, USA). The sample size was 200 µL. All results were the mean of three parallel samples (SD < 5%).

### 2.12. pH 6.8 Resistance Test

A capsule of EUDRACAP^®^ colon and HMPC, loaded with the 1:6:2 SD containing 10 mg of ABZ, which was used in the in vitro drug release studies, was placed in an adequate amount of pH 6.8 to test the EUDRACAP^®^ colon resistance to disintegration. This study aimed to demonstrate differences in release performance between the two capsule types by observing tartaric acid release from the solid dispersion. The indicator 0.2% *v*/*v* methyl red in ethanol 96% (Merck, Sigma-Aldrich Chemie GmbH, Taufkirchen, Germany) was used to visualize the dissolution of tartaric acid. The change in the colour of the capsule and the release medium (yellow at pH 6.8), turning red due to the release of TA from the capsules, was observed under the microscope at 25 °C (Keyence VHX-970F; lens VH-Z20R; Keyence Corp., Osaka, Japan; magnification ×20) and registered at predetermined time points over 30 min.

### 2.13. Test of Capsule Wall Permeability and Wettability of the Powder

The bottom part of an open EUDRACAP^®^ colon capsule, loaded with the 1:6:2 SD and the respective PM containing 10 mg of ABZ, was placed in an adequate amount of pH 6.8 buffer, up to the rim of the capsule. The medium contained 0.2% *v*/*v* methyl red indicator in ethanol 96% (Merck, Sigma-Aldrich Chemie GmbH, Taufkirchen, Germany). The powder within the capsule was observed under the microscope (Keyence VHX-970F; VH-Z20R; Keyence Corp., Osaka, Japan; magnification ×30), and images were taken at predetermined time points over 15 min. This study aimed to demonstrate performance differences between the spray-dried powder and the physical mixture by observing wettability and the concomitant dissolution of the powder in the capsule. As the powder mixture was wetted, tartaric acid started to dissolve, as evidenced by changes in the indicator’s colour in both the solid dispersion and the physical mixture.

To further demonstrate the different wettability performance of spray-dried powder compared to the physical mixture, the 1:6:2 SD and 1:6:2 PM powders were compressed into tablets with a Natoli NP-RD-10 tablet press using a round, flat-faced punch (7 mm diameter) at a compression force of 10 kN. The filling depth was set to 4.3 mm. The measured tablets’ hardness was 30 ± 5 N. An aliquot of 20 µL of phosphate-buffered solution (pH = 7.2) was added to the top of the tablet. The contact angle of the drop was observed under a microscope (Keyence VHX-970F; lens VH-Z20R; Keyence Corp., Osaka, Japan; magnification ×20, angle adjustment (90° tilt)), and images were captured at predetermined time points over 10 min. The drop was photographed and, therefore, the contact angle was analyzed and measured using the ImageJ (v.1.54g) software (National Institutes of Health, Bethesda, MD, USA) with the DropSnake plugin.

## 3. Results

### 3.1. Scanning Electron Microscope (SEM) Imaging Results

**Figure 2:**
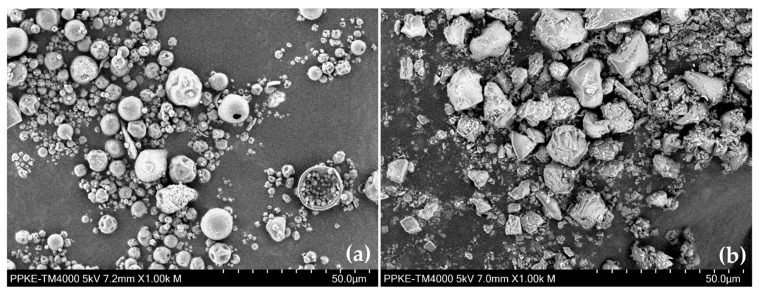
SEM of (**a**) 1:6:2 SD and (**b**) the respective PM.

SEM images of the spray-dried powder (SD) and its corresponding physical mixture (PM) are shown in Figure 2; the spray drying process produced generally spherical particles. The surface morphology of the spray-dried powders showed spherical, rigid and smooth particles (Figure 2a). Still, the majority of them show shrinkage and appear collapsed, with a non-rigid crust, which can be explained by the low outlet air temperature (30 ± 10 °C) maintained during spray drying. This phenomenon has been previously described [29]. In contrast, in Figure 2b, physical mixtures exhibit irregularly shaped, rough particles of larger size.

### 3.2. Thermodynamic Solubility

**Figure 3:**
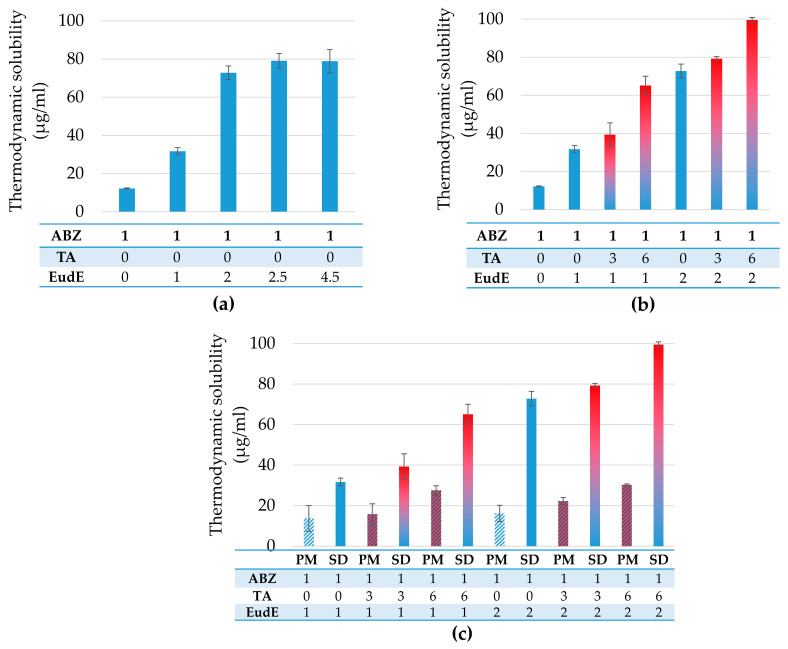
Thermodynamic solubility studies in pH = 6.8 of (**a**) ABZ-EudE SD with varying increasing ratios (1:0:0; 1:0:1, 1:0:2; 1:0:2.5 and 1:0:4.5), (**b**) ABZ-TA-EudE SD with varying ratios (1:0:1; 1:3:1; 1:6:1; 1:0:2; 1:3:2 and 1:6:2) and (**c**) comparison of SDs with respective PMs. The plain pattern corresponds to the spray-dried solid dispersions, the diagonal represents the corresponding physical mixture. The gradient colours indicate the relative ratio of TA (red) and, EudE (blue) in the samples.

To ensure comparable ionic conditions, thermodynamic solubility was determined at pH 6.8 (Ph. Eur. 11). The ionic strength of each buffer was calculated using Equation (3) and ranged from 0.10 to 0.12 M. According to the literature, these values correspond to the medium ionic strength range; therefore, differences in solubility cannot be attributed to substantial variations in ionic strength [30]. To maintain the consistent buffer strength, the thermodynamic solubility was determined at pH 6.8 (Ph. Eur. 11). The solubility of ABZ in solid dispersions with EudE is given in Figure 3a. The presence of EudE increased ABZ solubility to 72.80 ± 3.57 µg/mL, 79.04 ± 3.98 µg/mL, and 78.89 ± 6.07 µg/mL for 1:0:2, 1:0:2.5, and 1:0:4.5 SD, respectively, reaching the plateau concentration. The 1:2 ABZ-EudE ratio was chosen as the maximum ratio for further studies. The addition of TA further enhanced the solubility of ABZ-EudE SD, compared to binary ABZ-EudE formulations (Figure 3b). For example, the formulation ABZ-EudE 1:0:1 showed the maximum concentration of 31.70 ± 1.89, while the ternary formulation ABZ-TA-EudE 1:3:1 and 1:6:1 increased solubility to 39.32 ± 6.25 µg/mL and 65.13 ± 4.90 µg/mL, respectively, and in the case of 1:3:2 and 1:6:2 to 79.29 ± 1.03 and 99.53 ± 1.29 µg/mL, respectively, compared to 72.80 ± 3.57 µg/mL of 1:0:2 SD.

### 3.3. Statistical Evaluation of Thermodynamic Solubility Studies

A two-factor, three-level full factorial design and experimental data of the investigated solubility values are presented in Table 3.

**Figure 4:**
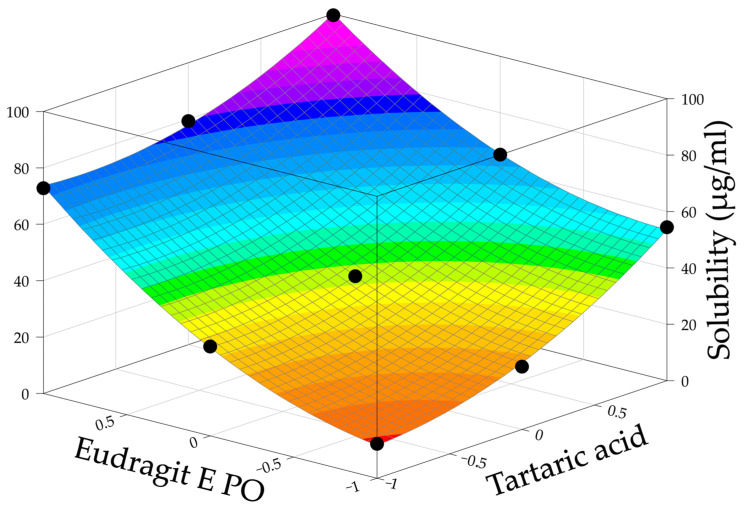
Effect of the excipient composition of SD samples on ABZ solubility. The response surface illustrates the polynomial model-predicted solubility as a function of Eudragit E PO and tartaric acid concentrations. The color gradient represents increasing predicted solubility from orange (low) to purple (high). Black dots indicate the experimental design points used to construct the model.

The resulting response surface of the experimental design is shown in Figure 4. The polynomial model provided a statistically significant fit at the 95% confidence level. The effect of two independent variables on the average solubility value is described by the following equation (Equation (5)):

(5) $$\mathrm{Solubility}\;\left(µg/\mathrm{mL}\right)\;=\;39.38\;+\;17.06x_{1}+\;27.11x_{2}+\;8.99x_{1}^{2}\;+\;11.37x_{2}^{2}-3.87x_{1}x_{2}$$

where the squared correlation coefficient (R^2^) is 0.999, and the fit standard error (root mean square error) is 1.43. The estimated coefficients and their significance are presented in Table 4.

Both variables, TA and EudE, have a significant positive effect on ABZ solubility: higher ratios lead to a higher solubility. In fact, the strength of the effect of TA:ABZ and EudE:ABZ is indicated with a positive sign (+17.06 and +27.11, respectively), implying that the increasing ratio of the respective excipient to ABZ increases the solubility. However, as shown in the surface plot (Figure 4) and in the magnitudes of the variables (+27.11 for EudE:ABZ and +17.06 for TA:ABZ), given in the function for b_1_ and b_2_ coefficients (Equation (3)), variable EudE has a more significant effect on the solubility (*p* = 0.00002) than TA (*p* = 0.00009). Furthermore, a quadratic effect is observed for both excipients, indicating that the relationship is not purely linear. The interaction effect (b_12_ = −3.87) is significant (*p* = 0.01228).

### 3.4. FT-IR

**Figure 5:**
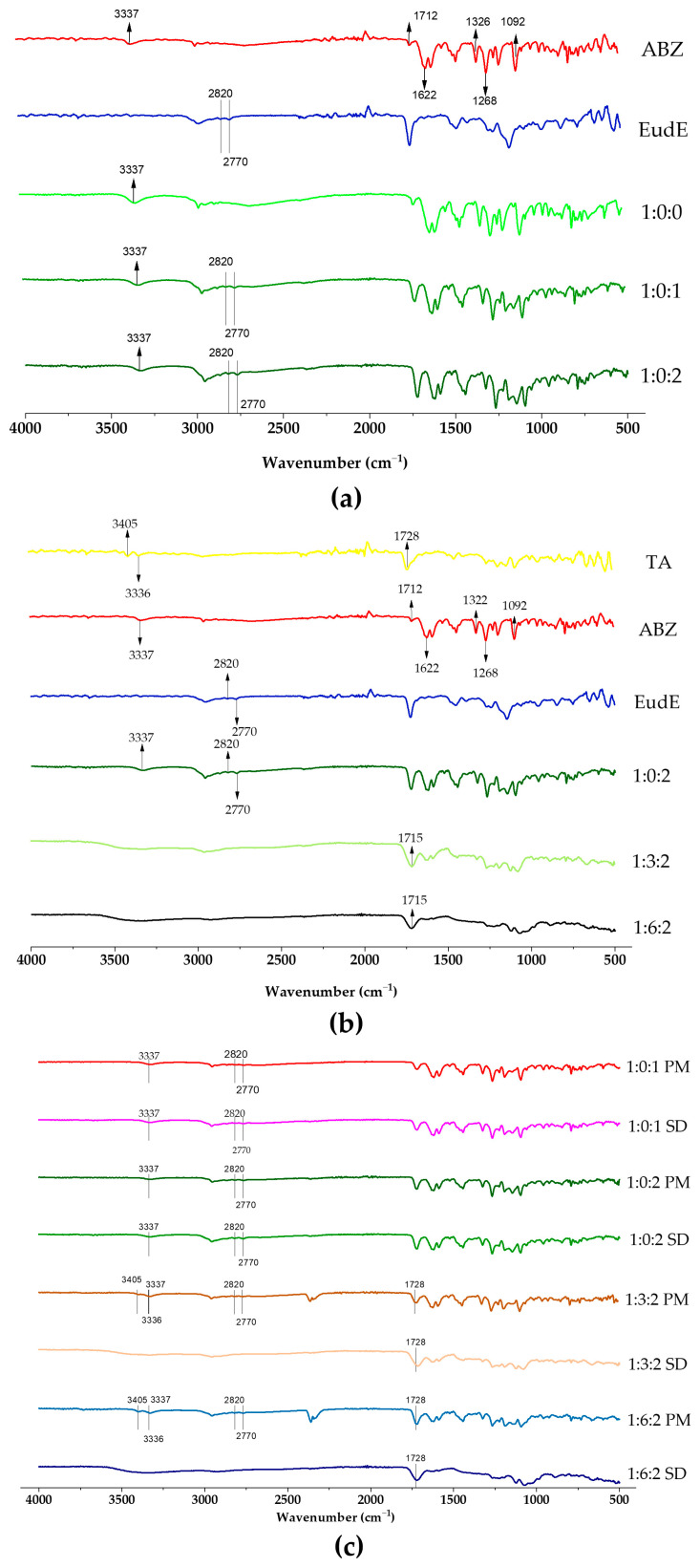
FT-IR spectra of ABZ-EudE solid dispersions in the (**a**) absence of TA, (**b**) presence of TA and (**c**) comparison of spray-dried solid dispersions (SDs) with the respective physical mixtures (PMs).

FT-IR spectroscopy was used to investigate potential specific interactions between the drug and the polymer, and between the drug, the polymer, and the organic acid. The spectra of crystalline ABZ, EudE and respective spray-dried solid dispersions containing ABZ and EudE in varying ratios are shown in Figure 5a to investigate the presence of eventual specific interactions between ABZ and EudE.

The most important peaks of crystalline ABZ are located at around 3337 cm^−1^ (N–H stretching), at 1712 cm^−1^ (C=O peak of carbamate), at 1326 cm^−1^ and 1268 cm^−1^ (aromatic C-N stretching and N–H bending), 1622 cm^−1^ (C=N stretching) and 1092 cm^−1^ (C–O stretching of the carbamate group). These characteristics of the ABZ FT-IR spectrum were previously described in the literature [6,10]. Similarly, these peaks were still present in solid dispersions with EudE, indicating that there are no significant specific intermolecular interactions between ABZ and EudE. The absence of specific interactions is further confirmed by the presence of the dimethylamino group band of EudE in the spray-dried solid dispersions of all the ratios of ABZ:EudE (found between 2820 and 2770 cm^−1^), identified as an indicator of interaction in a previous study [18].

In Figure 5b, the FT-IR spectra of solid dispersions in the presence of TA are reported. Conversely, the reported characteristic peaks of ABZ in spray-dried solid dispersions where both TA and EudE are present are more extensive and weaker, which can suggest an amorphization by the presence of TA. Furthermore, the broader peak of N-H stretching of ABZ and the movement of the carboxylic group stretching of TA from 1728 cm^−1^ to a lower wavelength in solid dispersions suggests the formation of a hydrogen bond between ABZ and TA in solid dispersions (Figure 5b). The O-H stretching of TA found between 3405 and 3336 cm^−1^ disappeared, which could also suggest that the carboxylic group of TA ionized into carboxylate anions that interact with the amino groups of ABZ through the hydrogen bond. Furthermore, the carboxylate anion can interact with the dimethylamino group band of Eudragit E, which is found between 2820 and 2770 cm^−1^. This group band disappeared in solid dispersions in the presence of TA, indicating that TA protonated this functional group. The same observation in the presence of TA was previously reported by Maghsoodi et al. [18].

The FT-IR spectra of the PMs and their respective SDs were compared to evaluate the effect of spray drying on the molecular interactions among the drug, polymer, and organic acid in the solid dispersions. In Figure 5c, it can be observed that the characteristic O-H stretching of TA found between 3405 and 3336 cm^−1^ is missing in the SDs 1:3:2 and 1:6:2, conversely from the physical mixtures. Furthermore, the N-H stretching of ABZ at 3337 cm^−1^ is found to be present in the SDs that do not contain TA and all PMs (1:0:1 SD and PM, 1:0:2 SD and PM, 1:3:2 PM and 1:6:2 PM), but is absent in SDs 1:3:2 and 1:6:2 containing TA. The carboxylate ion peak at 1728 cm^−1^ shifts to lower wavenumbers in SDs containing TA. Furthermore, in the SDs 1:3:2 and 1:6:2, the dimethylamino groups of the EudE band disappeared compared to the spectra of the physical mixtures. These spectral changes in SDs containing TA, compared with the physical mixtures and SDs that do not contain TA, indicate the formation of specific interactions during spray-drying in the presence of TA.

### 3.5. DSC

**Figure 6:**
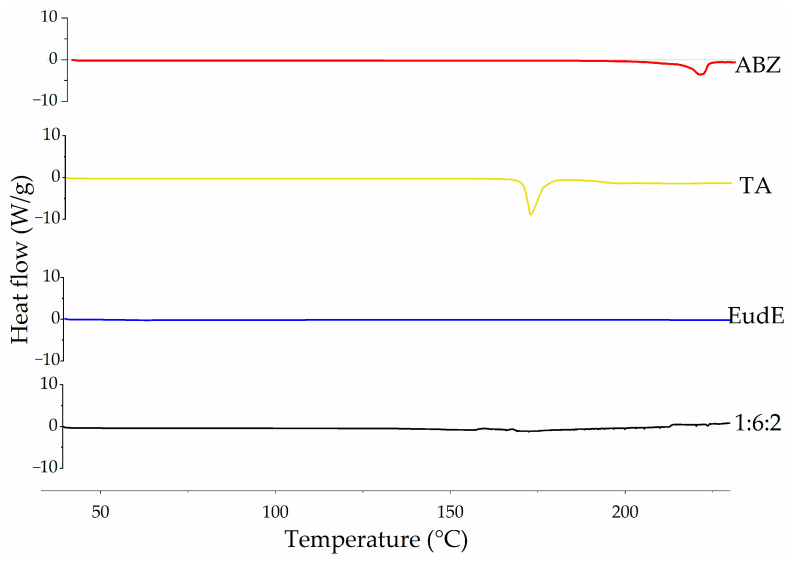
Differential scanning calorimetric thermograms of albendazole powder (ABZ), Eudragit E PO (EudE), tartaric acid (TA), and spray-dried solid dispersion 1:6:2.

Figure 6 shows DSC thermographs of ABZ powder, EudE, TA and spray-dried solid dispersion 1:6:2. ABZ powder displayed a distinct endothermic peak at around 220 °C, corresponding to its melting point. In comparison, the characteristic single peak between 170 °C and 180 °C corresponds to TA’s melting point. On the contrary, no characteristic peaks were observed for EudE, confirming its amorphous structure. Furthermore, no distinct peaks were observed in the DSC thermogram of the spray-dried 1:6:2 solid dispersion.

### 3.6. P-XRD

The ABZ diffractogram is consistent with previous results by Chattah et al. and Pranzo et al. [31,32] showing that ABZ powder consists of two polymorphic forms, Form I and Form II. The main characteristic peaks of Form I are identified at 6.9, 11.3, 11.6, 18, 24.4 and 27 (°2θ), while Form II peaks were also consistent with the literature and identified at 7.3, 10.7, 14.6, 18.1, 25 and 30 (°2θ).

**Figure 7:**
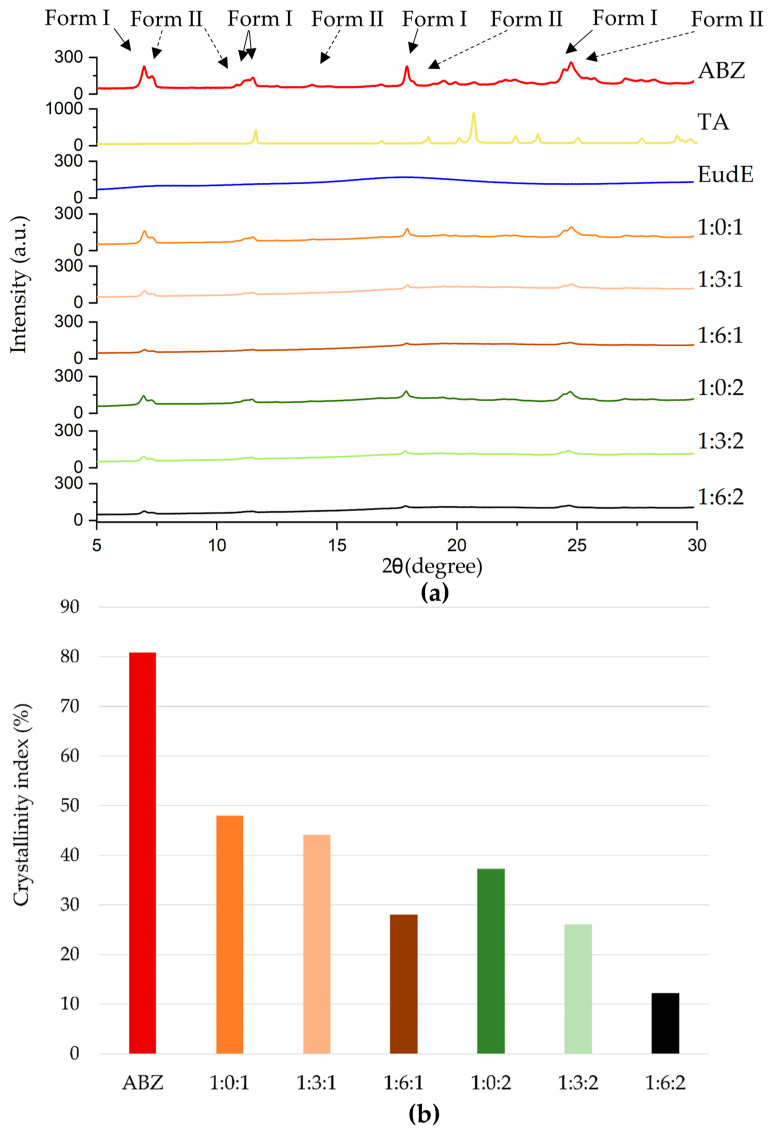
(**a**) P-XRD diffractograms of ABZ, TA, EudE and spray-dried solid dispersions 1:0:2, 1:3:2 and 1:6:2 and (**b**) crystallinity index (CI) of ABZ and the respective solid dispersions 1:0:2, 1:3:2 and 1:6:2.

The P-XRD measurements confirm the amorphous character of EudE and the crystalline structure of ABZ and TA. The latter shows sharp diffraction peaks, in contrast to EudE, which showed none (Figure 7a). The SD samples containing different ratios of the two crystalline components, along with the EuE, transformed into weak, broad halos in the 1:0:1, 1:3:1, 1:6:1, 1:0:2, 1:3:2, and 1:6:2 spray-dried solid dispersions, indicating a transition to a partly amorphous structure. The crystallinity index (CI) calculated using Equation (2) decreases with increasing TA content (Figure 7b), as confirmed by the broader peaks in Figure 7a. Previously, it was also described that the protonization of EudE by an acid increases the amorphousness of the sample [33].

### 3.7. In Vitro Drug Release Tests

The Weibull distribution semiempirical model function was fitted to the release profiles by minimizing the sum of squared residuals between experimental and predicted data points (Table 5). The lowest correlation between the fitted and measured values (0.9859) was observed for the EUDRACAP^®^ colon capsules filled with the physical mixture. The release of ABZ from the HPMC capsules was immediate, and due to its high solubility, it reached a plateau within a very short time (t_0_ = 0.1 h). The release of the EUDRACAP^®^ colon capsules filled with ABZ powder and the physical mixture of excipients is similar, as confirmed by the Weibull parameters. After a 4 h lag time, the 63.2% release defined by τ_D_ is reached in approximately 3 h.

**Figure 8:**
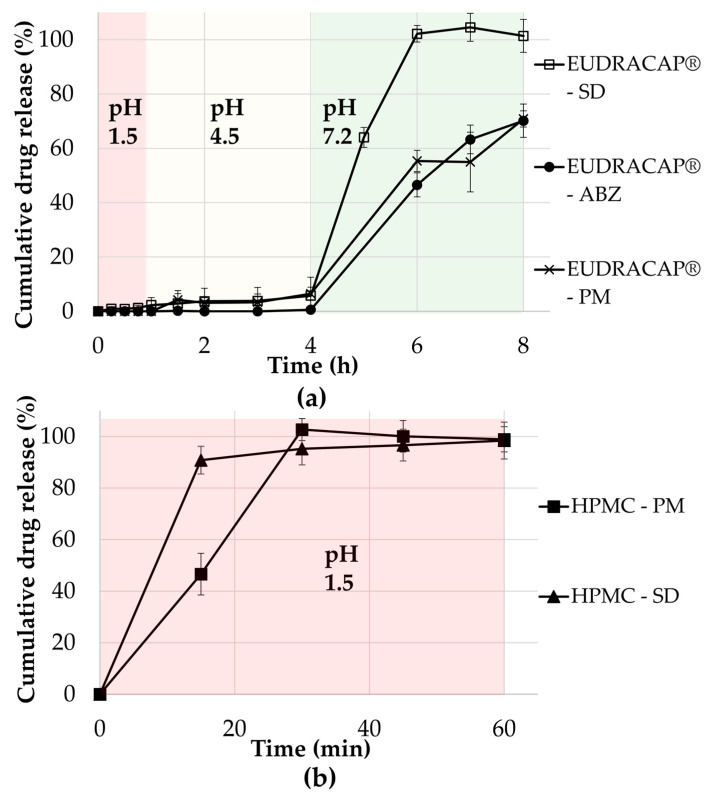
In vitro drug release of (**a**) ABZ, 1:6:2 solid dispersion and the respective physical mixture in EUDRACAP^®^ colon and of (**b**) 1:6:2 solid dispersion and the respective physical mixture in HPMC capsules.

The kinetics, as determined by Weibull fitting, follow first-order kinetics (β = 1). The SD powder, when filled into capsules with a protective coating layer that allows release only at higher pH, also shows a 4 h delay. However, the release will be more rapid, as confirmed by the shorter τ_D_ (τ_D_ = 1 h) and the shape parameter (β = 1.5), which are characteristic of a sigmoidal profile, indicating a more complex dissolution process. The ABZ release from the EUDRACAP^®^ colon was prevented at acidic pH and started only after 4 h, when the pH was changed to 7.2, which is attributed to the performance of EUDRACAP^®^ colon capsules (Figure 8a). The same dissolution-release performance of EUDRACAP^®^ colon-resistant in an acidic medium, compared to hard gelatin capsules, was observed by Agbaje et al. [34]. However, the release from the 1:6:2 solid dispersion reached 100%, whereas the release from the ABZ powder and the 1:6:2 PM was around 50% after 6 h (τ_D_ = 3 h after a 4 h lag-time). The release performance of ABZ powder and the 1:6:2 PM was similar, suggesting no increase in solubility for the physical mixture. In contrast, the ABZ release from HPMC capsules began at pH 1.5 and reached 100% cumulative drug release after 1 h (Figure 8b), in both the 1:6:2 SD and the respective PM. In the first 15 min, ABZ release from the spray-dried powder was faster, but after 15 min, both PM and SD reached maximum cumulative drug release due to ABZ’s high solubility at the acidic pH.

### 3.8. Microenvironmental pH (Micro pH)—By Slurry Method

**Figure 9:**
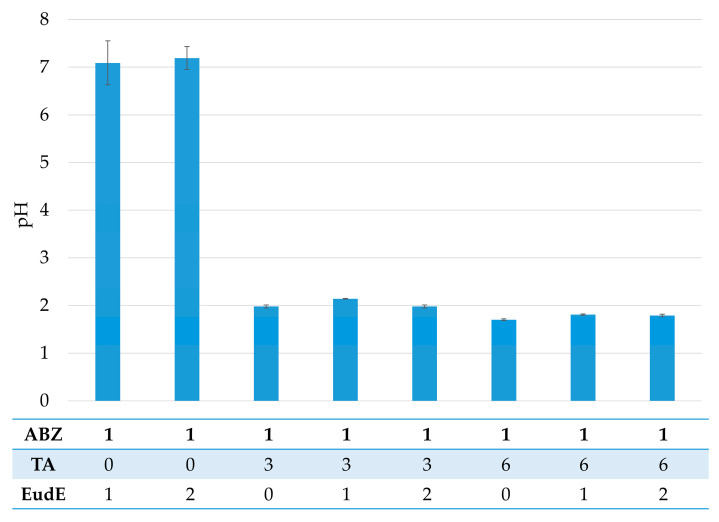
Microenvironmental pH studies conducted over 24 h in distilled water.

As shown in Figure 9, the microenvironmental pH decreases with the addition of TA to binary ABZ-EudE systems. The ABZ-TA-EudE slurries’ pH was 3–4 units lower compared to binary ABZ-EudE slurries. These results are consistent with the increased ABZ solubility observed in ternary formulations (Figure 3b), as ABZ exhibits pH-dependent solubility. The pH values across the ternary formulations were similar, indicating no significant difference in the microenvironment. However, slight differences in the microenvironmental pH can lead to significant changes in solubility [12].

### 3.9. Capsule Wall Permeability and Wettability of the Powder

**Figure 10:**
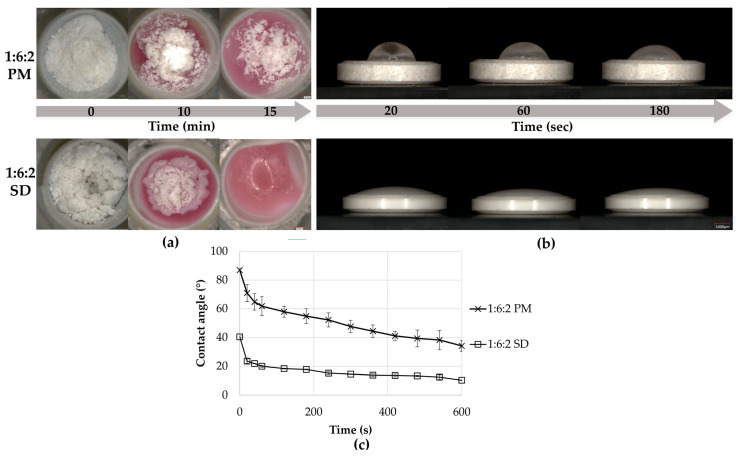
(**a**) Powder wettability (Keyence VHX-970F; lens VH-Z20R; Keyence Corp., Osaka, Japan; magnification ×30); red scale indicates 1000 µm (**b**) contact angle observation of a pH 7.2 droplet on the surface of the tablets from 1:6:2 physical mixture (PM) and 1:6:2 spray-dried (SD) powders and (Keyence VHX-970F; lens VH-Z20R; Keyence Corp., Osaka, Japan; magnification ×20, angle adjustment (90° tilt)); (**c**) contact angles measured by image analysis.

Figure 10a illustrates the wetting properties of the spray-dried formulation 1:6:2 and its corresponding physical mixture. As shown, after the same time frame (15 min), the solid dispersion exhibits better wettability. The lower contact angles (Figure 10b) confirm that the spray-dried powder has better wettability than the corresponding physical mixture. Contact angle measurements reveal that the spray-dried powder achieved a contact angle of 19.46 ± 0.38 at 60 s, considerably lower than the 61.79 ± 6.49 determined for the physical mixture (Figure 10c).

### 3.10. pH 6.8 Resistance

**Figure 11:**
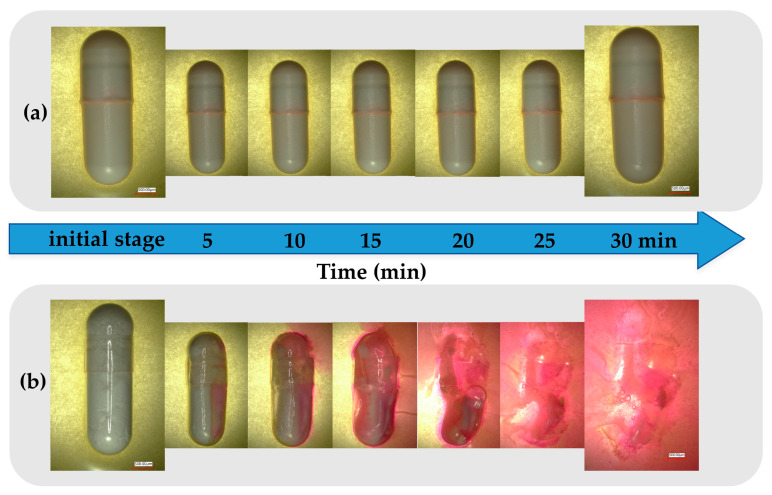
Tartaric acid release in the presence of methyl red indicator at pH 6.8 from (**a**) EUDRACAP^®^ colon and (**b**) HPMC capsules. Methyl red turns red, indicating the pH change from 6.8 towards an acidic pH due to the release of tartaric acid from the capsules. Scale bar shows 500 µm.

Tartaric acid release from EUDRACAP^®^ colon and HPMC capsules was tested over 30 min. It was observed that EUDRACAP^®^ did not release TA, confirming its resistance to disintegration at pH 6.8, as reported in the technical sheet provided by Evonik [35] (Figure 11a), differently from HPMC capsules, which disintegrated completely after 25 min, as reported previously in the literature [36] (Figure 11b).

## 4. Discussion

In the present work, we formulated EUDRACAP^®^ colon capsules containing EudE and pH-modifier-based spray-dried solid dispersions to deliver ABZ to higher pH and to improve its solubility. EUDRACAP^®^ colon capsules have previously been used for colon-delivery of nanosuspensions and niosomes [5,34]. EUDRACAP^®^ colon capsules are intended to protect drug release at lower pH in the upper gastrointestinal tract and to deliver the drug at pH 7.2 in the ileocolonic region [35]. The present findings confirmed this performance: as shown in Figure 8a, in vitro drug release from EUDRACAP^®^ colon capsules occurred only at pH 7.2, not at pH 1.5 or 4.5. The performance of EUDRACAP^®^ colon capsules was also tested at a slightly acidic pH. These capsules at pH 6.8 were stable; no TA release was observed in the in vitro test over the 30 min, as indicated by the absence of a noticeable red colour in the dissolution media (Figure 11a). In contrast, for the HPMC capsules, disintegration began within the first 5 min. It was completed in 25 min, as evidenced by the red colour due to the release of tartaric acid, which lowered the media pH (Figure 11b).

Spray-drying is a common technique used to induce amorphization of poorly soluble drugs and increase their solubility. Amorphization of crystalline drugs is an approach to increase the solubility of poorly soluble active pharmaceutical ingredients [37]. The amorphous form has a higher theoretical solubility compared to the crystalline form, because there is no energy needed to break the crystalline lattice [38]. EudE has previously been used to increase the solubility of poorly soluble drugs by amorphizing the active ingredient in a solid dispersion, thereby substantially increasing solubility and, consequently, bioavailability [39]. As shown in Figure 7b, there was only a partial amorphization (crystallinity index = 37.26% and 47.96% for 1:0:2 and 1:0:1, respectively) when EudE was used as a carrier in solid dispersion, because of a lack of specific interactions (Figure 5a), which were only formed by the addition of TA (crystallinity index = 12.16%, and 28.09%, in the case of 1:6:2 and 1:6:1 solid dispersions, respectively), causing the formation of specific interactions (Figure 5b). The increased specific intermolecular interactions formed in the ternary solid dispersions, as evidenced by FT-IR, were observed in the P-XRD spectra as a decrease in ABZ crystallinity compared to the binary solid dispersions (Figure 7a), where no specific interaction was observed by FT-IR (Figure 5a). Jambukiya et al. previously showed that as the organic acid content increases, crystallinity decreases, indicating increased amorphousness. The authors suggested that the amorphization led to a superior in vitro dissolution release, due to intermolecular interactions, thereby stabilizing the dissolution [10]. As evidenced by the aforementioned study, our study also confirmed that an increase in amorphous content in spray-dried solid dispersions containing higher tartaric acid led to a higher dissolution rate than in ABZ powder and the physical mixture (Figure 8a). The partial amorphousness of the sample 1:6:2 SD was further confirmed by the disappearance of the melting point of ABZ in the DSC spectra (Figure 6).

In addition to serving as a simple carrier in a solid dispersion, EudE directly influences drug solubility through intermolecular interactions, which can be either hydrophilic or hydrophobic [40]. In the case of acidic drugs, such as mefenamic acid, the solubility enhancement of the drug-EudE solid dispersion compared to the respective physical mixture is given principally by the hydrophilic types of interaction (ionic or hydrogen bond) between the polymer and the mefenamic acid [41]. However, Higashi et al. showed that, besides hydrophilic interactions, which were considered the most significant intermolecular interactions responsible for the solubility increase, the hydrophobic interactions between the aromatic hydrophobic rings of mefenamic acid and the backbone C-CH_3_ of EudE also contributed to the drug-polymer interaction and therefore to the stabilization of the supersaturated solution of the solid dispersion and consequent bioavailability [41].

EudE is a cationic copolymer of methyl metacrylate, N-N dimethyl aminoethyl metacrylate, and butyl metacrylate monomers, containing a tertiary amino group, which gets ionized at acidic pH, making it highly soluble at pH lower than 5 [13,42]. Therefore, due to EudE’s cationic character, it is expected to form hydrophilic interactions (ionic or hydrogen-bonding) with acidic drugs, as mentioned previously. However, in addition to increasing the solubility of acidic drugs through hydrophilic interactions, EudE also increases the solubility of weakly basic drugs, despite the drugs and the polymer having the same positive charge. The intermolecular interactions that contribute to the increase in the solubility are therefore purely hydrophobic [21]. As shown in Figure 3a, the solubility of ABZ powder increased from 12.21 ± 0.24 µg/mL to 31.70 ± 1.89 µg/mL and to 72.80 ± 3.57 µg/mL in the presence of EudE in 1:0:1 and 1:0:2 spray-dried formulations, respectively. By increasing the EudE ratio, the solubility reached a plateau; as the polymer concentration increased, solubility did not increase further, as observed by Saal et al. [21]. The solubility increased due to possible nonspecific hydrophobic interactions between the hydrophobic part of the drug (aromatic ring) and EudE. To support this hypothesis, FT-IR spectra of 1:0:2 ABZ-EudE (Figure 5a) show no change in peaks (shift or new peaks) compared to pure materials, which suggests the absence of ionic or covalent interactions between drug and polymer.

To further increase ABZ solubility, a pH modifier, TA, was added to promote specific interactions in the ternary drug-pH modifier-polymer system by providing a localized acidic microenvironment [10]. Ueda et al. previously showed that adding a ternary component, saccharin, to API-EudE increased drug release [43]. Furthermore, Fung et al. showed that adding an organic acid to the polymer-basic drug binary system of ketoconazole increased the API dissolution rate [44]. The incorporation of TA increased the solubility of API-polymer systems (Figure 3b). As previously suggested by Mild et al., TA can increase the solubility of EudE-based solid dispersions by two mechanisms: by providing acidic microenvironmental pH and by the protonation of EudE that was maintained by localized microenvironmental acidic pH [12], which was confirmed by the acidic microenvironmental pH of solid dispersion slurries (Figure 9). In the solid dispersions containing TA, the characteristic bands of dimethylamino groups of EudE were no longer detectable, as they were protonated by TA, suggesting a specific ionic interaction (Figure 5b). The protonation of EudE can increase electrostatic repulsion between the basic API and EudE, thereby promoting drug release [12]. The increase in solubility due to a tartaric acid-induced decrease in pH was previously reported [12,45].

Furthermore, as shown by FT-IR (Figure 5b), TA interacted with ABZ by forming hydrogen and/or ionic bonds, which was already noticed previously in ABZ-organic acid solid dispersions [10]. Previous studies showed that the bonding interaction of organic acids with API contributed to the stabilization of the active ingredient by reducing the molecular mobility of the drug and increasing API solubility in ternary systems, compared to API-polymer binary systems [44]. The addition of TA to the binary ABZ-EudE system caused an enhancement in ABZ amorphization (Figure 7a). As shown in Figure 7b, the crystallinity index decreased with the addition of TA, further confirming the FT-IR results and indicating specific interactions between ABZ and TA (Figure 5b).

Statistical evaluation of thermodynamic solubility studies (Figure 4) showed that both the pH modifier and the polymer have significant effects on solubility (*p* = 0.00002 and 0.00009, respectively). Still, EudE has a larger effect than TA (b_2_ = 27.11 and b_1_ = 17.06, respectively). Compared with the effects of separate components, the interaction between the two factors was less significant (*p* = 0.01228). It showed a negative coefficient (b_12_ = −3.87) (Table 4), indicating that the contemporaneous increase in EudE and TA has a lower effect on the solubility of ABZ than an increase in either variable alone [46].

To demonstrate the advantages of the spray drying process for increasing solubility, the physicochemical characteristics of physical mixtures and in vitro drug release were compared with those of spray-dried solid dispersions. In Figure 3c, it can be observed that spray-dried solid dispersions exhibit higher solubility in a pH 6.8 medium than the corresponding physical mixtures. Fung et al. previously suggested that the increased solubility of the co-amorphous API-acid-polymer system was achieved through the morphology and size of the spray-dried particles and the microenvironmental pH provided by the organic acid [11]. SEM images show significantly different morphologies for spray-dried and physical mixtures’ particles (Figure 2). Sphericity and a more uniform particle size of spray-dried particles (Figure 2a) can significantly influence the powder’s wettability performance [47], and better wetting performance can increase the in vitro drug release of solid dispersions [48]. A previous study by Alasino et al. examined the interfacial behaviour of EudE in solution. It showed that EudE exhibits significant surface activity, which depends on solution pH, decreasing at lower pH [49].

The advantage of spray-drying in the powder’s performance was further confirmed by the in vitro drug release studies (Figure 8). In the case of EUDRACAP^®^ colon (Figure 8a), release occurs only in a slightly basic pH, where ABZ solubility is low. Therefore, the solid dispersion prepared by spray drying strongly determines drug release. The cumulative drug release from the spray-dried formulation reached 100% at 6 h, whereas the physical mixture reached 50%, indicating a significant increase in drug release for the solid dispersion (Figure 8a,b). A higher drug release rate was observed for spray-dried powders compared to physical mixtures, due to their better wettability (Figure 10a,b). The improved wettability can be explained by better morphological characteristics of the spray-dried powder (Figure 2a). Furthermore, previous studies showed that polymers used as carriers in solid dispersions can improve the wetting properties of hydrophobic drugs compared to the physical mixtures [50]. Wettability can significantly increase the dissolution rate of a powder, as previously reported in studies [51,52,53]. Spray-dried samples in the capsule showed better, quicker wettability in the first 15 min than the physical mixtures (Figure 10a), which can explain the higher dissolution rate observed in the in vitro drug release studies at alkaline pH. Furthermore, the contact angles of compressed tablets with the spray-dried powders were lower than those of the corresponding physical mixtures (Figure 10b,c), further confirming the spray-dried powders’ better wettability. Conversely, the ABZ release from HPMC capsules did not differ significantly between the spray-dried and physical mixtures (Figure 5b), which can be explained by ABZ’s high solubility at acidic pH (Figure 8b).

The advantage of the spray-drying process was further confirmed by FT-IR spectra comparing SDs with PMs containing TA, indicating that the specific interactions between the drug, polymer, and tartaric acid form only during the spray-drying process (Figure 5c).

Our in vitro results provide preliminary insights into the performance of the colon-delivery formulation at different physiological pH values. However, for the prediction of in vivo performance of the proposed formulation, different factors that can influence the drug release should be taken into consideration, such as: inflammatory bowel diseases and consequent pH of the colon [54], colon transit [55] and a diet rich in fibre, which is fermented into short-chain fatty acids by the microbiota [56].

## 5. Conclusions

This study shows that both tartaric acid (TA) and Eudragit E PO (EudE) significantly improve the solubility of albendazole (ABZ) in solid dispersions prepared by spray drying. Adding TA, as a pH modifier, to EudE-based solid dispersions creates an acidic microenvironment, thereby increasing specific interactions within the solid dispersions. The spray-dried solid dispersions were evaluated for drug release and physicochemical characteristics, showing better performance than physical mixtures and ABZ powder. Functional ready-to-fill capsules EUDRACAP^®^ colon loaded with solid dispersions prevent drug release in the upper parts of the gastrointestinal tract and deliver the drug at higher pH, characteristic of the ileocolonic region.

The formulation selected for capsule filling was identified based on its superior performance, exhibiting the highest solubility, the greatest extent of amorphization and favourable specific and non-specific interactions in an ionically crosslinked system. The study demonstrated that EUDRACAP^®^ colon capsules filled with spray-dried solid dispersion released ABZ at higher pH, with improved solubility. Therefore, the study managed to couple spray-drying’s ability to improve drug solubility with intermolecular drug-polymer-organic acid interactions, while enabling targeted ABZ delivery through the application of functional ready-to-fill capsules.

To predict the in vivo performance of the proposed formulation, further studies are required, as multiple inter- and intra-individual factors influence the colonic area.

## Figures and Tables

**Table 1:** Compositions per weight (g) of the total solid content of ABZ-loaded solid dispersions and physical mixtures.

Mass Ratio of Components/ Component Name	1:0:0	1:0:1	1:0:2	1:0:2.5	1:0:4.5	1:3:0	1:6:0	1:3:1	1:6:1	1:3:2	1:6:2
ABZ	2	2	2	2	2	2	2	2	2	2	2
TA	0	0	0	0	0	6	12	6	12	6	12
EudE	0	2	4	5	9	0	0	2	2	4	4

**Table 2:** Experimental setup, independent variables (x_1_ and x_2_) and their respective values included in the experimental design.

	Actual Values	
Coded Value	x_1_, Organic Acid TA (TA Mass Ratio to ABZ)	x_2_, Polymer EudE (EudE Mass Ratio to ABZ)
−1	0	0
0	3	1
+1	6	2

**Table 3:** Two-factor, three-level full factorial design codes and results (mean standard deviation, *n* = 3).

Sample Number	Ratio of the Components (ABZ:TA:EudE)	x_1_	x_2_	Solubility (µg/mL)
1	1:0:0	−1	−1	12.21 ± 0.24
2	1:0:1	−1	0	31.70 ± 1.89
3	1:0:2	−1	1	72.80 ± 3.57
4	1:3:0	0	−1	22.30 ± 1.06
5	1:3:1	0	0	39.32 ± 6.25
6	1:3:2	0	1	79.29 ± 1.03
7	1:6:0	1	−1	54.44 ± 7.71
8	1:6:1	1	0	65.13 ± 4.90
9	1:6:2	1	1	99.53 ± 1.29

**Table 4:** Summary of statistical analysis of the thermodynamic solubility values.

Model F Value	Parameter	Coefficient					
		b_0_	b_1_	b_2_	b_11_	b_22_	b_12_
650.80 (*p* = 0.00009)	Value	39.39	17.06	27.11	8.99	11.37	−3.87
	Standard error	1.06	0.58	0.58	1.01	1.01	0.71
	*p* > |t|	0.00004	0.00009	0.00002	0.00298	0.00150	0.01228

**Table 5:** Weibull model parameters estimated by nonlinear least-squares minimization of the sum of squared residuals.

	HPMC	EUDRACAP^®^ Colon		
	Solid Dispersion	ABZ Powder	Physical Mixture	Solid Dispersion
**M_∞_ (fixed)**	**100**	**100**	**100**	**100**
**t_0_ (fixed)**	0.0	4.0	4.0	4.0
**τ_D_**	0.1	3.0	3.0	1.0
**β**	1.0	1.0	1.0	1.5
**correlation**	0.9991	0.9990	0.9859	0.9974

## Data Availability

The original contributions presented in this study are included in the article. Further inquiries can be directed to the corresponding author.

## Abbreviations

The following abbreviations are used in this manuscript:

ABZ	Albendazole
API	Active pharmaceutical ingredient
ASD	Amorphous solid dispersion
BCS	Biopharmaceutical classification system
DSC	Differential scanning calorimetry
EudE	Eudragit^®^ E PO
FT-IR	Fourier transform infrared
HPMC	Hydroxypropyl methylcellulose
PM	Physical mixture
P-XRD	Powder X-ray diffraction
SD	Solid dispersion
SEM	Scanning electron microscopy
TA	Tartaric acid
